# Successful management of allergy to the insulin excipient metacresol in a child with type 1 diabetes: a case report

**DOI:** 10.1186/1752-1947-6-263

**Published:** 2012-08-31

**Authors:** Benjamin J Wheeler, Barry J Taylor

**Affiliations:** 1 Department of Women’s and Children’s Health, University of Otago, Dunedin, 9054, New Zealand; 2 Edgar National Centre for Diabetes and Obesity Research, University of Otago, Dunedin, 9054, New Zealand

## Abstract

**Introduction:**

Insulin allergy to human insulin preparations during the treatment of diabetes is suggested to occur at rates ranging from <1.0% to 2.4%. These reactions vary from mild localized reactions, which resolve with repeated exposure, to life-threatening anaphylaxis and death. The management of persistent insulin allergy in type 1 diabetes mellitus is particularly complicated because ongoing treatment with insulin is essential.

**Case presentation:**

We present the case of a 12-year-old Caucasian girl with localized allergy to the insulin excipient metacresol, and the subsequent desensitization therapy using continuous subcutaneous insulin infusion with simultaneous intravenous insulin infusion.

**Conclusions:**

This is the first documented case of allergy to the metacresol component of insulin in the pediatric type 1 diabetes literature. We describe an approach to diagnosis and management of metacresol allergy in type 1 diabetes.

## Introduction

Insulin allergy, although less common since the introduction of human insulin [1], is still an issue in the management of diabetes. Suggested rates of insulin allergy range from <1% to 2.4% [1,2], covering the spectrum from mild localized reactions, which resolve with repeated exposure [3], to life-threatening anaphylaxis or death [4]. The management of persistent insulin allergy in type 1 diabetes mellitus (T1DM) is particularly complicated because ongoing treatment with insulin is essential. Commercially available insulin contains multiple ingredients: the insulin molecule itself in a variety of forms; and a range of additives, preservatives and buffers, commonly referred to as excipients [5]. Narrowing down the specific cause of the allergy is therefore not simple. In the past, this has been aided by the commercial availability of insulin allergy test kits [5-7], however, manufacture of these has recently ceased. We present a case of allergic reaction to the insulin excipient metacresol in a child with T1DM, and describe a diagnostic approach and management.

## Case presentation

**Figure 1:**
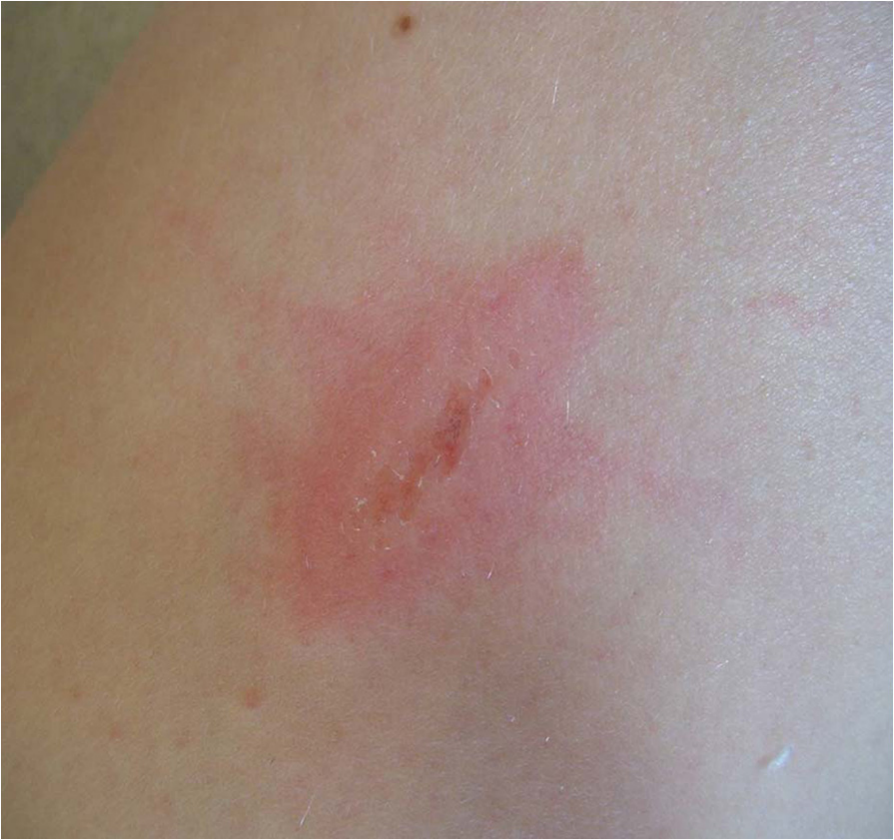
Immediate localized skin breakdown.

**Figure 2:**
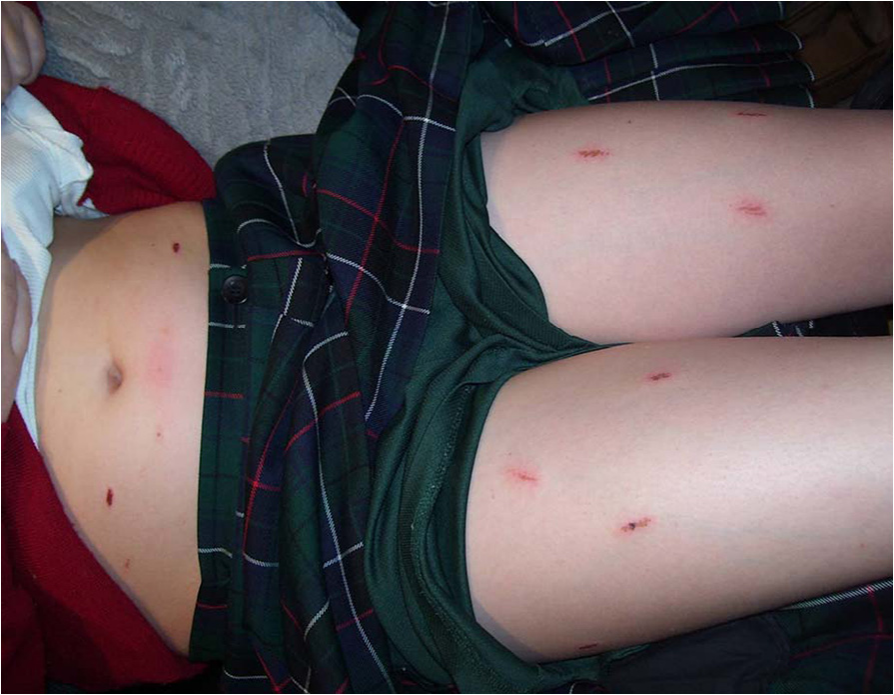
Multiple healing abrasions.

A 12-year-old Caucasian girl with newly diagnosed T1DM was commenced on twice-daily basal Protaphane® and bolus insulin aspart with meals. Within the first week of treatment she complained of increasing pain with Protaphane® injections. By Week 2, this had progressed to include additional localized erythema. Humulin neutral protamine Hagedorn (NPH)® was substituted, with some benefit but her symptoms persisted. Glargine and detemir insulins were then trialed but localized symptoms were worse. Past history revealed a localized erythema to some soap and adhesive preparations. At 6 weeks from diabetes diagnosis, her aspart injections also began to cause pain and localized erythema. This progressed over a few weeks to include localized skin breakdown occurring within 5 minutes of injection (Figure 1) that left her with multiple healing abrasions (Figure 2). The pain and distress were such that glycemic control was impaired because of injection avoidance. Antihistamines (loratidine and cetirizine) resulted in some reduction in her Humulin NPH®-related symptoms, but with no effect on those of aspart.

Negative responses were obtained from subcutaneous testing with latex, and following injections of 0.9% saline with a variety of skin preparations and needle types.

Initial blood work revealed negative results for specific immunoglobulin E (IgE) to: human insulin, porcine insulin, bovine insulin, and latex. Eosinophils were 0.2 (reference range <0.9). Total IgE was raised at 112kU/L (reference range <100).

Next, subcutaneous testing was conducted on all available insulin preparations (Protaphane®, Actrapid®, lispro, aspart, glulisine, glargine, Humulin NPH®, Humulin R®, detemir). All elicited positive responses: pain followed by localized erythema, urticaria, and eventual rapid skin breakdown. An allergic reaction to human insulin seemed less probable because the human insulin molecule differs between preparations. To explore this fully, Novo Nordisk® diluting medium, which does not contain insulin but does have similar excipients to insulin aspart, was the next step. The subcutaneous test with Novo Nordisk® diluting medium elicited a similar positive reaction.

Because the patient had an allergic reaction to all available insulin, a review of insulin preparations and excipients was conducted as described by Heinzerling *et al*. [5]. Metacresol was the only excipient common to all but it was not available commercially for testing. However, on review we discovered that the only significant ingredient in Lilly™ ‘saline’ penfills was metacresol. Subcutaneous testing again elicited an identical positive reaction. Thus metacresol was the only agent common to all positive tests. Based on this finding, a presumed allergy to the excipient metacresol was diagnosed.

Desensitization therapy was commenced 6 weeks from first presentation using insulin aspart via continuous subcutaneous insulin infusion (CSII). Intravenous Actrapid® via a peripherally-located central catheter was used to maintain normoglycemia and prevent ketosis. This was well tolerated at infusion rates ≤3 units/hour. Rates above this resulted in central chest discomfort. CSII was commenced at a rate of 0.025 units/hour with a plan to increase the infusion rate by 0.05 units every 6 hours. If the patient felt minor discomfort, then the rate would remain unchanged, but if she had significant pain or urticaria the rate was to be reduced by 0.05 units/hour. Within 2 hours of commencement the infusion was stopped due to immediate localized urticaria followed by substantial pain. At this stage, 3 days of oral prednisone 20mg was added. On Day 2, a tenfold dilution with 0.9% saline was used to reduce the starting dose of aspart to 0.0025 units/hour. A pump rate of 0.25 units/hour was reached without incident, and a twofold aspart dilution was substituted at a new pump rate of 0.05 units/hour (0.025 units/hour aspart). When a pump rate of 0.3 units/hour was tolerated, undiluted aspart was substituted and commenced at 0.15 units/hour. The estimated full basal rate of 0.35 units/hour was successfully achieved by 120 hours. At this point, meal boluses were added, initially with a slow wave meal bolus over 4 hours. This was replaced at 156 hours with a dual-wave bolus (50% immediate, 50% over 2 hours). By 168 hours, small (<3.5 units) standard boluses of aspart were tolerated without discomfort, increasing to 6.5 units by 312 hours.

## Discussion

We describe the first documented case of an allergic reaction to the metacresol component of insulin in the pediatric T1DM literature. Allergy to the metacresol in insulin has been described previously in an adult with T1DM [8]. Because metacresol is universally present in all current insulin preparations, we believe it has been overlooked as a possible cause of insulin allergy in some past case reports. The specific nature of the skin reaction, in our case erythema, urticaria, pain, and excoriation with abrasion and/or epidermal separation within 5 minutes of injection, is unusual and not previously described. It is probable that the skin reaction represents a mixed allergic response with Type I and IV allergy components. The wider literature on metacresol describes burn and skin breakdown reactions with cutaneous exposure [9], which may help explain this phenomenon. Metacresol is present in a variety of common products ranging from soaps to adhesives, agents to which our patient had experienced mild reactions in the past. A dose-response relationship was also observed: the lowest reaction was seen with Humulin NPH® (metacresol 1.6mg/mL) and the most severe reactions were seen with lispro and glulisine (metacresol 3.15mg/mL).

In the past, the diagnosis of insulin allergy was facilitated by commercially available insulin allergy test kits [5]. These kits contained all the potential ingredients of commercial insulin preparations including several types of insulin and a range of excipients that included preservatives (e.g. metacresol), retardants (e.g. protamine sulfate), stabilizers (e.g. zinc), acid and base buffers, and isotonic agents (e.g. glycerol). Recently, manufacture of these kits has ceased with the current literature not responding to this change in circumstance.

We describe a novel approach to this dilemma. Common preparations are available that can be used via subcutaneous testing, in conjunction with blood tests, to isolate a specific cause of insulin allergy after excluding other common causes, for example injection technique, reaction to latex, skin preparations or needle types. The procedures to start a specific investigation are twofold: blood tests, including total IgE and specific IgE to human, porcine and bovine insulin as well as latex; and subcutaneous testing of all available insulin preparations. The taking of antihistamines during the testing and three days prior to testing is to be avoided. Because the active ingredient (insulin) and some excipients do differ between preparations, the pattern of reaction might suggest the cause. Next, Novo Nordisk™ diluting fluid can be helpful to distinguish an excipient allergy from true insulin and/or aspart allergy. Because the Novo Nordisk™ diluting fluid does not contain insulin but does have similar excipients to insulin aspart, a negative result would strongly suggest an allergic reaction to aspart and not to an excipient, whereas a positive result would indicate an excipient allergy. Investigating metacresol, the preservative universally present in available commercial insulin, is the next step. Lilly™ ‘saline’ for practice pen injection contains metacresol as the only ingredient other than sodium chloride, water for injection, and acid/base buffers. A positive reaction to subcutaneous testing with Lilly™ ‘saline’ strongly suggests metacresol allergy.

If diagnosed, options for treatment of non-spontaneously resolving metacresol allergy are few. Past insulin preparations did not contain metacresol, for example some porcine insulins, Monotard®, and Ultratard®. However, metacresol is present as a preservative in all currently available insulin, making a desensitization approach essential. Traditionally, successive subcutaneous injections of increasingly less dilute preparations of insulin are given [5]. More recently, SCII has been used with success. In previous case reports, [6,10] a low basal rate of between 0.1 units/hour and 0.3 units/hour has been successful at initiating desensitization; however, as we describe, substantially lower concentrations or even dilution with sterile saline may be required.

The time required for successful desensitization varies, and is patient and technique specific. If titration proves slow, then ketoacidosis from insulin deficiency quickly results. Intravenous insulin as previously described [10,11] can provide an avenue for temporary insulin replacement during desensitization. Why intravenous therapy as opposed to subcutaneous therapy is generally well tolerated is not fully understood. Suggested mechanisms range from the simple mechanics of putting small volumes of insulin into a large central vein with subsequent rapid distribution (particularly relevant for localized reactions) through to differences in the immune system response depending on the route of insulin administration [11]. Complications can still occur, with transient urticaria documented [10] along with the novel central chest pain seen in our patient.

Systemic treatment with oral antihistamine and/or steroid has been used [12]. We found twice-daily oral antihistamine provided minimal improvement. Oral steroids possibly provided some benefit during our desensitization therapy, but are not ideal as a long-term option. Our patient continues on an antihistamine, but for subsequent local flare-ups we have had success using soluble hydrocortisone 0.1mL (50mg/mL) added to 1.9mL aspart insulin in the pump reservoir, to provide low-level local immune suppression.

## Conclusions

In this report we document the first case of allergy to metacresol in the pediatric diabetes literature; metacresol is an excipient common to all currently available insulin preparations. To the best of our knowledge this is also the first documented case of metacresol allergy successfully treated with desensitization therapy. A novel and simple method of exploring the etiology of insulin allergy is described using readily available preparations. Although far from ideal, this method can allow the causative agent to be distinguished: whether this is human insulin, or an excipient.

## Consent

Written informed consent was obtained from the patient and the patient’s legal guardian for publication of this case report and accompanying images. A copy of the written consent is available for review by the Editor-in-Chief of this journal.

## Competing interests

The authors declare that they have no competing interests.

## Authors’ contributions

BW conceived and wrote the manuscript, as well as managed the patient from diagnosis through to treatment. BT was a major contributor in writing the manuscript, and assisted in the diagnostic process and its conception. All authors read and approved the final manuscript.
